# Conservative Surgery

**Published:** 1908-08-22

**Authors:** 


### The Hospital

A Journal of

The Medical Sciences and Hospital Administration.

New Series. No. 76, Vol. III. [No. 1148, Vol. XLIV.] SATURDAY, AUGUST 22, 1908.

### CONSERVATIVE SURGERY.

It is almost a truism that the advent of anæsthesia and the antiseptic *régime* has allowed the practice of operative surgery to fall into many hands not essentially suited to this most delicate of all handicrafts. Some degree of technical skill is picked up by every man before he becomes qualified; small local hospitals and "homes" afford abundant opportunities; and within the well-known restrictions of surgical cleanliness the human body will survive even dreadful injuries inflicted with the best intentions in the name of surgery. Far be it from us to forget or underrate the excellent work that is done in these institutions throughout the kingdom, or to hint that they alone are open to these charges. We wish, however, to draw attention to the conservative spirit which is developing more and more in the surgery of the great teaching hospitals throughout this and other countries. The term "conservative" does not by any means imply a reluctance or unreadiness to operate; it implies all that tends to preserve from loss, waste, or injury. Left severely alone, nephrolithiasis sometimes arrives at cure by suppuration and extrusion of the stone; but it would be a dangerously false interpretation of the function of conservative surgery which waited for this happy event to occur in any given case. Nor, in these days when even nephritis and emphysema have been brought into the fair field of surgical intervention, when vessels are sutured and there is prospect of successful transplantation of organs and even of joints, will it be supposed that conservatism implies any diminution of the vigour and boldness with which new methods are prosecuted. We are watching now with the deepest interest the development of what the Americans call the "clinical surgeon," one who is versed not only in the modern refinements of bedside diagnosis but also in all those accessory methods which physiological and pathological advances have laid open to him. No man now operates upon diseased kidneys at haphazard; the resources of physics, physiology, and mechanics have provided him with means for estimating the functional capacities of the kidneys together and severally. No man now excises a tuberculous joint or amputates a limb for a similar condition until the methods afforded by the new understanding of infective processes and the normal means of resistance have been exploited to the full. And if the time has not arrived when we can say quite as much for cancer, yet everyone knows that the most strenuous efforts are being made to bring that also within the range of possibility.

Whilst we have learnt that hardly any inflammatory process can for practical purposes be considered apart from the presence of micro-organisms, we have learnt also that bacterial activities may be inhibited and a recessive influence be exerted at a stage hitherto thought out of the question. Hyperæmia induced *secundum artem* will nowadays forestall the scalpel; we have returned to the habit of pre-antiseptic days and evacuate large collections of tuberculous detritus through an aspirating needle; the surgeon feels to-day that almost every amputation he performs is a reproach to his surgery; perhaps in an excess of zeal he even anticipates the dangers of infection now and then by protective inoculations before certain operations. So that whilst the impunity with which operations are performed may have tended to irresponsibility in submitting patients to the knife, modern surgery is essentially conservative; the surgeon does not look upon each patient as an opportunity to operate, but as an opportunity to display his skill in avoiding the knife. In the South African campaign and in the Russo-Japanese war it was demonstrated that aseptic wounds of the abdominal viscera and of the brain did extremely well on the routine of masterly inactivity, but that experience has not for one moment hindered the progress of civil practice in these fields; it has rather helped us to a better appreciation of the powers of the peritoneum and meninges to take care of themselves.

The "early operation" in appendicitis may be taken as a good example of the best conservative surgery; it is calculated to preserve the patient from loss, waste, and injury. The radical cure of hernia is conservative; it is not that a truss may not be effective, but that in a very great many cases it fails at precisely the times when most needed, and, moreover, that the flesh is weak and the truss a nuisance—it does no good in the bottom drawer. An operation for flatfoot may be distinctly conservative; three weeks' total inactivity is a small price to pay for freedom from a small discomfort that nevertheless may detract 30, 40, or 50 per cent. from the wage-earning capacity. The surgery which removes the thoracic portion of the greater pectoral, or maybe the whole muscle and the lesser pectoral as well, in operating for carcinoma of the breast is strictly conservative; it preserves all that is safely to be preserved for working purposes. The removal of an injured but "exciting" eye is conservative in the best sense. So, too, an open operation to screw a fracture of the neck of the femur in a young or middle-aged man is similarly to be characterised.

Conservative surgery, then, is something more than that which strives to save every quarter of an inch of every finger that may survive after a bad crush of the hand. The true conception of conservatism is wider and more comprehensive. It has as its aim the prophylactic or definitive preservation of every functioning part that promises to contribute to, and not detract from, the capacity of the individual to earn his livelihood and to enjoy life. One little word of caution may be uttered. There is a tendency to overlook or ignore the duration of the effects of all considerable operations. A perfectly successful appendicectomy will often leave a man for several months with his capacity for work and his general activity appreciably diminished; or, again, an operation for the radical cure of hernia may be undertaken without previously ascertaining that the patient can abstain from heavy work for the length of time necessary to give the operation a chance of the complete and durable success properly to be expected from it. Progress in pathology and surgery is broadening the basis of surgical therapeutics; its employment in the modern conservative spirit demands, whilst it also fosters, the confidence of our patients.

